# Health Anxiety by Proxy Disorder: A Case Report

**DOI:** 10.7759/cureus.34058

**Published:** 2023-01-22

**Authors:** HusamEddin Z Salama, Yousef A Alnajjar, Mousa O AbuHeweila, Osama N Dukmak, Ibrahim Ikhmayyes, Shafee Saadeh

**Affiliations:** 1 School of Medicine, Al-Quds University, Jerusalem, PSE; 2 Psychiatry, Dr. Kamal Psychiatric Hospital, Bethlehem, PSE

**Keywords:** worries, parents, illness anxiety, hypochondriasis, psychotherapy, cognitive behavioural therapy (cbt), by proxy, health anxiety

## Abstract

Health anxiety by proxy (HAP) is a newly introduced term in psychiatry to describe the anxious feelings or fear of having or acquiring a serious illness. It is often accompanied by maladaptive illness behavior in the absence of true somatic symptoms. This, in turn, entails seeking medical advice and therefore doing many unnecessary investigations in an attempt to justify these symptoms. Functional impairment may appear in HAP patients, and this indicates a pathological point. To some extent, it can be said that HAP is similar to health anxiety disorder in terms of symptomatology and items. However, it is imposed on another (usually the patients’ children) instead of the patient himself. Many biopsychosocial factors are suggested to play a role in the psychopathology of HAP. Until now, there are no well-established criteria in the Diagnostic and Statistical Manual of Mental Disorders, Fifth Edition, Text Revision (DSM-5-TR) or International Classification of Diseases 11^th^ Revision (ICD-11) to make the diagnosis of health anxiety by proxy. Although treatment protocols are missing, it appears that patients are not responding to treatment protocols for illness anxiety disorder. This requires focusing attention on conducting studies on those patients to develop clear treatment plans to help patients. In this report, we present a 28-year-old female with constant worries about her child’s health, which subsequently resulted in seeking medical advice at multiple clinics with different medical specialties. Many factors were thought to be implicated in triggering her current condition. The anxious feelings reflected negatively on the patient's life, resulting in a poor functioning status. A treatment plan was initiated with a dismal response and fluctuating course. Additionally, we discussed the initial definition and the bases that could be used to facilitate the diagnosis and management of HAP disorder.

## Introduction

Illness anxiety disorder (IAD), previously known as hypochondriasis, is classified as a somatic symptom and related disorder in the Diagnostic and Statistical Manual of Mental Disorders, Fifth Edition (DSM-5). It is characterized by rumination and intense worries about harboring serious illness [1]. This condition is often triggered by a misunderstanding of one's own bodily sensations [2], which are explained as alarming signs of a serious illness and associated with maladaptive illness behavior with recurrent body examination and/or medical advice seeking [3], which mostly provides transient relief of their anxiety [4].

DSM-5 describes two types of IAD: the care-seeking type and the care-avoidant type. The care-seeking type involves those who constantly use the healthcare system, including physician visits and performing multiple tests and procedures. On the other hand, the care-avoidant type refers to patients who avoid seeking medical advice. They have severe anxiety and believe their physician visit or laboratory test will reveal life-threatening illnesses like cancer [5].

Clinical observations suggest that illness anxiety may extend to affect others, such as children. In comparison to healthy women, recent studies have shown that mothers who suffer from illness anxiety disorder would display higher levels of maladaptive health behaviors towards their children. Health anxiety by proxy (HAP) has recently been introduced as a term to describe excessive parental concerns and preoccupation with their child's symptoms and fear that physicians will overlook a serious disease in their child [6]. Consequently, this will expose the child to unnecessary and potentially unpleasant medical examinations and procedures.

HAP has been largely neglected in pediatric settings [6]. Therefore, till 2021, no standardized measures for systematic assessment were established. In 2021, a group of researchers developed the Health Anxiety by Proxy Scale (HAPYS) to systematically assess HAP [7]. Twenty-six items characteristic of HAP has been introduced in the last update of this scale. The current study aims to expand our knowledge and understanding of this type of somatic symptom and related disorder. Herein, we present a case of a care-seeking type of health anxiety by proxy, whose disease course has been characterized by recurrent visits to the physician to seek reassurance about her son's health in the past five years with no resolution of signs and symptoms.

## Case presentation

The patient has a bachelor's degree in psychology but does not have a job. She married eight years ago and has three sons aged seven years, four years, and 15 months. During her lifetime, she went through many biopsychosocial stressors, the most prominent of which was that her marital relationship was not interconnected or enjoyable. She claimed that her marital relationship was problematic, with multiple ups and downs. She also mentioned that her husband always neglects her, and she is the only one who puts much effort into this relationship. She also mentioned a history of sexual abuse when she was young.

The patient presented to the clinic complaining of constant worry about her youngest child's health for the past year. This started when she noticed a spot on her son's back, which, in turn, raised her suspicion that her son might suffer from a serious illness. Subsequently, the patient began to search and investigate the matter and visited many doctors from different specialties to check on her son's health. Her visit to the physician used to give her a transient relief of her anxiety. However, gradually, she began to increase the frequency of her physicians' visits with minor relief of her symptoms. At first, she used to seek medical attention about three times a month. Later on, her disease started progressing, and thus, her physicians' visits increased. She mentioned that she went to four physicians in one day. At the time of initial medical seeking, she had been told that her son might have McCune Albright syndrome. Therefore, her concern became centered and preoccupied with that illness.

During the five-months before coming to the psychiatric clinic, she visited most of the medical specialties, the most prominent of which were: Pediatricians, Dermatologists, Neurologists, Endocrinologists, and Orthopedists. All the physicians, after conducting some necessary examinations and labs, agreed that her son's health was good and he did not suffer from any diseases. However, this did not reassure her and did not change her view on anything, which encouraged one of her relatives, who primarily works in the medical field, to advise her to visit a mental health clinic.

Accordingly, after attending the psychiatrist, a treatment plan was started. The patient was first started on escitalopram 20mg for three months. In the first few weeks of drug therapy, she showed slightly elevated mood, talkativeness, and to a small extent, slight relief in her anxiety. Later on, her response started to decline, and the patient relapsed despite the increase in drug dose. Thus, venlafaxine 75mg twice daily and propranolol 10mg PRN was tried for one month. During this period, the patient's condition worsened, and she began to have suicidal thoughts and tried to commit suicide using medications. She also mentioned that she started to worry about her older child's health status due to the emergence of a new spot on his back. Thus, propranolol has been replaced with risperidone 1mg twice daily. There was minor improvement related to poor inter-family support and fluctuated husband's attention. Accordingly, the patient started on fluoxetine in addition to the previously mentioned drugs. It is important to mention that the patient underwent several cognitive behavioral therapy (CBT) sessions in conjunction with medications throughout her treatment period.

With those medications, the patient slightly improved with a fluctuating course. She mainly did better with psychotherapy, especially in the first few days of the treatment, in addition to family support and attention. However, most of the time, the patient showed the same concerns related to her son, anxiety, health behaviors, and poor functionality. She recently started to avoid showing her family her anxiety related to her suspected son's illness. To exemplify this, she mentioned that she took her son to outpatient clinics multiple times without telling her family to avoid their worry about her condition.

## Discussion

Health anxiety by proxy disorder (HAP) is characterized by excessive worries about having or developing a serious and undiagnosed disease in someone else (usually the patient's child's health) [6]. These worries and concerns persist despite normal physical examination and investigations. Physical symptoms are not present, and if they are present, they are minimal. As health anxiety by proxy is described as a new phenomenon in mental health, there are no epidemiological studies about this new phenomenon. Therefore, any case that raises suspicion for this disorder should be reported to help develop diagnostic criteria and management for this newly defined disorder.

The clinical presentation of HAP is suggested to be similar to that of an illness anxiety disorder (IAD). However, the concern will be on someone else's health, such as the patient's child, as presented in this case, instead of the patient's health. Symptoms of illness anxiety occur on a spectrum from physiological to pathological and are considered pathological when anxiety about health causes functional impairment, as demonstrated in the case presentation [8,9]. Patients often discuss their illness concerns with others, investigate the suspected diagnoses, and seek reassurance from others.

These preoccupations are heterogeneous, so patients may be preoccupied with a particular diagnosis, alteration in body function, or vague concern. The patient's concern may involve one or more organ systems, and the concern may shift over time from one organ or disease to another [10]. It is apparent from the patient history that the disorder has a chronic course with fluctuating symptoms.

The preoccupation with the feared illness often becomes a central focus of the patient's life, which leads to high healthcare utilization. Moreover, sometimes patients may press the physician to do invasive diagnostic tests and higher-risk treatments. Seeking care from multiple clinicians, "doctor-shopping" is prominent in this case, supporting the diagnosis. Comorbid psychopathology may be present, but it is often missed due to attention on the primary disorder.

According to DSM-5 and DSM-5-TR, there are no diagnostic criteria for HAP. Therefore, we diagnosed this disorder according to DSM-5-TR criteria applied for IAD but with the concerns imposed on the patient’s child [11]. The patient is preoccupied with her child having or developing a serious illness in the setting of mild or absent somatic symptoms. Substantial anxiety about her child’s health was evident, with a low threshold for becoming alarmed about his condition. There were reported excessive health-related behaviors manifested by repeatedly visiting multiple physicians and doing extensive investigations. The preoccupation was for more than six months (about one year in our case) and is not explained by other medical or mental disorders such as generalized anxiety disorder or somatic type of delusional disorders.

A newly designed scale measures excessive parental worries about children's health. Health Anxiety by Proxy Scale (HAPYS) is designed to provide a systematic assessment of health anxiety by proxy. The final version of this scale consists of 26 item characteristic of health anxiety by proxy and an impact section with five items [7]. Although this scale measures excessive parental worries but does not set a definitive way to diagnose health anxiety by proxy disorder. In the future, after creating definitive characteristics for diagnosis, this scale can be an important tool to diagnose this disorder.

General medical disorders should be ruled out before diagnosing health anxiety by proxy disorder. Additionally, symptoms of health anxiety by proxy disorder may overlap with the symptoms of other psychiatric disorders, and sometimes, it is difficult to make a diagnosis. The differential diagnosis for HAP may include one of these: normal reactions, adjustment disorder, factious disorder imposed on another (Munchausen syndrome by proxy (MSP)), generalized anxiety disorder (GAD), obsessive-compulsive disorder (OCD), specific phobia, panic disorder, psychotic disorders, unipolar major depression or persistent depressive disorder (dysthymia). 

Patients with health anxiety by proxy disorder have excessive concerns and preoccupations that are unrelated to symptoms' severity and persist for at least six months in contrast to normal reactions. On the other hand, patients with adjustment disorder may have anxiety and worries about their children's health. However, adjustment disorder is a diagnosis of exclusion. It occurs within three months of an onset of an identifiable non-life-threatening stressor and lasts ≤ 6 months following the resolution of that stressor. Meanwhile, patients with MSP intentionally falsify symptoms or induce injury in someone else. Both are not present in this case. Patients with GAD may have worries about their children's health, but they are also preoccupied with worries about other aspects of their lives, making this diagnosis unlikely.

Patients with HAP may have intrusive thoughts about their children's health and develop compulsive behaviors (e.g., visiting physicians). However, in HAP, the preoccupations usually focus on having a disease. In contrast, in obsessive-compulsive disorder (OCD), the thoughts are intrusive and usually focus on fears of getting a disease in the future. Additionally, patients with OCD seek reassurance in an attempt to relieve or reduce anxiety associated with the intrusive thoughts related to their children's health, while patients with HAP visit physicians in a try to find the diagnosis of their children's illness and manage them accordingly.

HAP patients are primarily preoccupied with issues about someone else's health, not specific objects or situations; thus, the diagnosis of a specific phobia is unlikely. They may develop panic attacks like those with panic disorder, but these attacks are triggered by concerns about the child's health. In contrast, in panic disorder, these attacks are not limited to health concerns. They also understand that their concerns and fears may not be true, as opposed to patients with psychotic disorders who have intense and rigid beliefs associated with other signs of psychosis (e.g., hallucinations or disorganized speech). Patients with major depression or persistent depressive disorder may not accept reassurance to their concerns regarding their children's health, especially during their major depressive episodes. However, if their worries and concerns persist despite the remission of their depressive episode, the diagnosis of HAP should be considered. Additionally, they also have other symptoms (e.g., insomnia, anorexia, anergia, impaired memory and concentration, and suicidality) [11].

The most important aspect of the management of IAD is establishing a strong therapeutic alliance with the patient [12]. It is essential for the patients to feel that their concerns and worries are understood and not underestimated by the physician. This could be achieved by regular visits to the same physician or psychiatrist to have an open dialog and monitor how their fears and concerns improve or worsen over time [13]. Medical and psychiatric management of patients with IAD tends to improve coping with fears and worries rather than eliminate them. This is done to prevent patients from adopting the sick role and becoming chronically invalid, thus ignoring their real medical issues [14,15]. The goal of treatment for those patients is to relieve their health worries by reassuring them that medical illnesses are ruled out based on medical and laboratory investigations to limit their visits to specialists and avoid unnecessary diagnostic procedures. This is also done to reduce the wasting of medical resources and physicians’ time and efforts. Some studies estimated that about 20% of the medical budget in the United States is spent on patients with some form of somatization or IAD [16].

The management of IAD is mainly done by psychotherapy, pharmacotherapy, or a combination of both. There are no head-to-head trials to compare the effect of using a combination to using either of the treatments alone. Still, some studies suggest that psychotherapy is superior to medications. Thus, nowadays, psychotherapy is considered the first-line treatment for patients with hypochondriasis [17]. Many studies showed that cognitive behavioral therapy, behavioral stress management, and psychoeducation effectively improved patients’ health-related thoughts and behaviors. It also helped in the relief of their anxiety and depression [18].

The use of medications such as antidepressants is usually considered a second line of treatment or an alternative to psychotherapy in cases of poor accessibility to CBT or in cases of patient denial of psychotherapy [19]. Additionally, we can use the medications as a first-line treatment in case of IAD with other comorbidities like depressive disorders. Antidepressants such as selective serotonin reuptake inhibitors (SSRIs) and serotonin-norepinephrine reuptake inhibitors (SNRIs) have been proven to be effective in patients with IAD. Additionally, patients should be reassured and given detailed information about the plan of treatment to enhance their compliance because many of them may misinterpret the use of medications as an attempt to ignore their worries.

Despite the use of multiple lines of treatment, from psychotherapy to different drug regimens for managing our patient, her disease course showed mild improvement with multiple relapses. This suggests that the management of health anxiety by proxy should not be similar to that of the usual IAD. Thus, we need to develop a new protocol to improve the outcomes of those patients in the future.

## Conclusions

Health anxiety by-proxy describes the preoccupation with excessive worries about other person’s health, especially patients’ children, in the absence or minimal somatic symptoms. It is essential for the patients to feel that their concerns and worries are understood and not underestimated by the physician. This can ensure that those patients will receive the proper treatment and avoid the unnecessary treatment of their healthy relatives. Until today, there are no definitive criteria to diagnose patients with this disorder, but it seems to be similar to that of usual IAD. The management of HAP seems to be similar to IAD, where psychotherapy and antidepressants are the mainstays of treatment. However, they are not as effective, which suggests the need for new protocols for the management of HAP.
